# Population Structure of Barley Landrace Populations and Gene-Flow with Modern Varieties

**DOI:** 10.1371/journal.pone.0083891

**Published:** 2013-12-27

**Authors:** Elisa Bellucci, Elena Bitocchi, Domenico Rau, Laura Nanni, Nicoletta Ferradini, Alessandro Giardini, Monica Rodriguez, Giovanna Attene, Roberto Papa

**Affiliations:** 1 Dipartimento di Scienze Agrarie, Alimentari ed Ambientali, Università Politecnica delle Marche, Ancona, Italy; 2 Dipartimento di Agraria, Università degli Studi di Sassari, Sassari, Italy; 3 Consiglio per la Ricerca e Sperimentazione in Agricoltura, Cereal Research Centre (CRA-CER), Foggia, Italy; CNR, Italy

## Abstract

Landraces are heterogeneous plant varieties that are reproduced by farmers as populations that are subject to both artificial and natural selection. Landraces are distinguished by farmers due to their specific traits, and different farmers often grow different populations of the same landrace. We used simple sequence repeats (SSRs) to analyse 12 barley landrace populations from Sardinia from two collections spanning 10 years. We analysed the population structure, and compared the population diversity of the landraces that were collected at field level (population). We used a representative pool of barley varieties for diversity comparisons and to analyse the effects of gene flow from modern varieties. We found that the Sardinian landraces are a distinct gene pool from those of both two-row and six-row barley varieties. There is also a low, but significant, mean level and population-dependent level of introgression from the modern varieties into the Sardinian landraces. Moreover, we show that the Sardinian landraces have the same level of gene diversity as the representative sample of modern commercial varieties grown in Italy in the last decades, even within population level. Thus, these populations represent crucial sources of germplasm that will be useful for crop improvement and for population genomics studies and association mapping, to identify genes, loci and genome regions responsible for adaptive variations. Our data also suggest that landraces are a source of valuable germplasm for sustainable agriculture in the context of future climate change, and that *in-situ* conservation strategies based on farmer use can preserve the genetic identity of landraces while allowing adaptation to local environments.

## Introduction

Landraces are heterogeneous plant varieties that are reproduced by farmers as populations that are subject to both artificial and natural selection. Landraces are distinguished by farmers who usually gave them a name that is associated with their specific traits (in cereals, these often refer to the spike and kernel characteristics), and different farmers might use the same landrace with the cultivation of different populations. Thus, in a landrace, the diversity is structured between and within populations (at the field/farmer level). The within population component of the genetic diversity of landraces has been described in many studies using qualitative and quantitative traits, as well as molecular tools (for review, see [1]).

Over the last 100 years, modern plant breeding has led to the development of elite cultivars that have often been based on a single genotype (pure lines, hybrids or clones) with improved yield potential, quality, and pest resistance traits [2]. The process of agricultural industrialisation was associated with the loss of many of the landraces that were reproduced on farms through farmer selection of seed for the planting of the next generation. In Europe, landraces were replaced by modern varieties starting from the 1920’s [2], [3], even if the widespread introduction of modern varieties took place only after the end of World War II. Moreover, in some marginal areas, the cultivation of landraces still continues, where farmers can take advantage of the specific adaptations of these landraces to the agro-ecosystem, for the preparation of typical products, and for cultural and religious reasons [4], [5].

Conservation of the genetic diversity of landraces can be static or dynamic [1], where static conservation is aimed at preserving the genetic identity of accessions that are usually conserved in gene banks. In contrast, dynamic conservation has the main target of preserving the evolutionary dynamics, with specific focus on adaptive processes, and this can be realized with the cultivation of plant populations both *in situ* and *ex situ* [1], [6].

The dynamic conservation of landraces is considered to be very important in the preservation of crop diversity in the centre of origin [7], as well as in areas of secondary diversification [5], [8]–[10]. In most cases, landraces are grown in co-existence with modern cultivars, and potentially with genetically modified varieties.

The occurrence of gene flow from modern varieties might constitute a menace to their genetic identity and diversity, because of unidirectional introgression from improved uniform varieties into the landraces [5]. Indeed, the varieties are reproduced in isolation by seed companies, and thus the gene flow should be acting only from modern varieties into landraces. A limited number of studies have estimated the gene flow between improved varieties and landraces; to the best of our knowledge, all of these were conducted in maize, which has an allogamous breeding system (e.g., [5], [11]). At the European level, the only gene flow analysis over time that has involved landraces and varieties was reported by Bitocchi *et al.* (2009) [5]. This study investigated introgression from modern hybrid varieties into landrace populations of maize, including ‘old’ and recent maize landrace collections that spanned 50 years, together with improved lines. In parallel, the aim of the present study is to provide information about the population structure, and gene flow and introgression between modern varieties and landraces with an important autogamous crop: barley.

Barley (*Hordeum vulgare* L.) is the fourth most important cereal worldwide in terms of grain production and area harvested (faostat.fao.org; last accessed, January 2012), and it is a strictly autogamous species (2n = 2x = 14) with an outcrossing rate of <1% [12], [13]. Barley is one of the first crops that were domesticated in the fertile crescent about 10,000 years ago [14]. Now, barley is grown worldwide, and its cultivation has spread over a wide range of agro-climatic conditions, with its production used for animal feed, as well as for human consumption, both directly and following its malting.

In Europe, the cultivation of barley landraces nowadays is limited to a few restricted areas, such as Sardinia in Italy, where their cultivation was documented by Attene *et al.* [4] along with their genetic diversity, adaptation to the local environment, and relevant interest as a source of germplasm for plant breeding [15]–[17].

In the present study, we compared the diversity of 12 populations of Sardinian barley landraces from two collections that spanned 10 years (in 1990 and 1999, for a total of 357 individuals) with a large and representative collection of modern barley varieties that were released between 1960 and 1998. The 59 modern varieties were chosen to represent the most widespread barley varieties used in Italy, and included types representative of the major barley germplasm subdivisions [18]–[20]: two-row and six-row types (this main barley germplasm subdivision is based on the fertility of the spikelets arranged in triplets and alternating along the rachis), and spring, winter and alternative types (based on vernalisation requirements). Three six-rowed lines (Micuccio, Zingaria, Pattyan) that have genotypes in their pedigree that were extracted from Italian landrace populations as progenitors were also included in the 59 modern varieties, to test the power of the method used in the present study for the detection of the effects of introgression.

We used simple sequence repeats (SSRs), with the main goals being to compare the Sardinian barley landraces with the pool of 59 modern varieties, in terms of their levels of gene diversity, and to determine the extent of gene flow and introgression that has occurred between these modern varieties and the Sardinian barley landraces. Considering that in Sardinia the spread of modern barley varieties started in the 1980’s, we would expect that if introgression from varieties into Sardinian landraces has taken place, there will be higher similarities between varieties and populations collected in 1999 than with those collected in 1990. The SSR loci used in the present study were tested for neutrality, to take into account the role and effects of selection and genetic sweep, to infer population structure and introgression, based on the putative neutral dataset obtained.

## Materials and Methods

### Plant Materials

We analysed 12 Sardinian six-rowed landrace (SL) populations of *Hordeum vulgare* L. (known as “S’orgiu sardu” by local farmers) that were collected on the island of Sardinia (Italy), a region that is characterised by a typical Mediterranean climate (see Table 1). For each barley population, we analysed 30 individuals (with the exception of SIS3 [28 individuals] and N2 [29 individuals]), for a total of 357 genotypes (Table 1). Six of the 12 SL populations were collected in 1990, from different agro-ecological areas of Sardinia (the 1990 SL population; SL90) for a total of 177 individuals, and the remaining six SL populations were collected during 1999 in the same geographical areas (the 1999 SL populations; SL99) for a total of 180 individuals. The individuals were randomly sampled from each field (one spike per plant). No permits were required for the described collection.

**Table 1:** The 12 Sardinian landrace (SL) populations used in this study (ordered by latitude).

Code	Name	Sample size	Year of collection	Location	Latitude	Longitude	Altitude (m.a.s.l)
COR	Corraxi	30	1999	Campidano	39°17′51″	9°07′01″	95
VI	Sestu	30	1990	Campidano	39°22′00″	9°06′00″	105
SAF	Sant'Andrea Frius	30	1990	Trexenta	39°28'50''	9°10'	300
SEN	Senorbi	30	1990	Trexenta	39°31′55″	9°07′02″	200
NXM	Nuraxi Mannu	30	1999	Trexenta	39°32′50″	9°05′06″	190
STU	Sturru	30	1999	Trexenta	39°36′07″	9°07′47″	374
SOR	Sorbacci	30	1999	Ogliastra	39°41′54″	9°34′34″	110
PIR	Pischina Rubia	30	1999	Sinis	39°52′01″	8°32′28″	35
SIS3	Sinis Sud 3	28	1990	Sinis	39°58′00″	8°32′01″	20
ORO	Orosei	30	1990	Baronia	40°19′50″	9°41′56″	240
N2	Nurra 2	29	1990	Nurra	40°43′50″	8°29′20″	85
CUM	Cuggia Manna	30	1999	Nurra	40°49′42″	8°33′27″	50

Among the 59 modern barley varieties (VAR) included in this study, 20 were two-rowed (Var2), as 11 spring and 9 winter types, and 39 were six-rowed (Var6), as 37 winter and 2 alternative types (Table S1). For each of these varieties, we analysed a single individual. Table S1 gives the pedigree information for each of the varieties, together with their country and year of origin. Three six-rowed varieties included in this study were considered as controls: Micuccio, Zingaria and Pattyan. Indeed, these three six-rowed varieties have pedigrees that include individuals extracted from local Italian populations.

### Molecular Data

Twelve SSR markers were selected from [21] and [22] and used to characterise the 416 individuals considered (Table 2). For the SL dataset, only 11 SSRs were polymorphic, and these were used for the subsequent analysis involving this dataset. DNA extraction and SSR genotyping were performed as described in [23]. PCR analyses were performed in a total volume of 20 µL, which contained 20 ng genomic DNA template, 50 pmoles of each primer, 200 µM dNTP, 2 mM MgCl_2_, 1× Taq polymerase buffer, and 0.5 U Taq DNA polymerase (Promega, Madison, WI, USA). One of the two SSR primers was end-labelled with 6-FAM or HEX (ABI Prism 3100-Avant Genetic Analyser). Amplifications were carried out with a Perkin-Elmer 9700 Thermal Cycler (PE Applied Biosystems, Foster City, CA, USA), with an initial denaturation of 3 min at 94°C, which was followed by 30 cycles of 1 min at 94°C, 1 min at X °C, and 1 min at 72°C, plus 10 min of final extension at 72°C (X °C refers to the annealing temperatures specified for each primer pair reported in Table 2, and to the touch down amplification, where the temperature decreased by 1°C every cycle, until the final annealing temperature of X °C. This was then followed by 26 cycles of 1 min at 94°C, 1 min at X °C, and 1 min at 72°C, plus 10 min of final extension at 72°C).

**Table 2:** Characteristics and genetic diversity parameters estimated for the 12

Marker name	Marker code	LG	cM	Forward primer	Reverse primer	Repeat motif	Reference	Reference molecular weight (bp)	Source of marker	QTL or geneproduct	Annealing temperature (°C)	*n_a_*	*n_e_*	*I*	*H_o_*	*H_e_*	*Fis*	*R_s_*
**Bmac0273**	BC	7H	93	ACAAAGCTCGTGGTACGT	AGGGAGTATTTCACCCTTG	(AC)_20_(AG)_20_	Ramsay *et al.* (2000)	186	Genomic DNA libraries	QTL abiotic stress	55	4.00	1.68	0.75	0.00	0.40	0.99	3.22
**HVMSIP1A**	HA	3H	N.D.	AACAATGTGTACGTCCACGC	ATTCCACACAAGCAACATTCAG	(CTT)_5_	Becker and Heun (1995)	149	Barley genes	Seed imbibition protein	65–60*	2.00	1.87	0.66	0.01	0.46	0.97	2.00
**Bmag0009**	BI	6H	103	AAGTGAAGCAAGCAAACAAACA	ATCCTTCCATATTTTGATTAGGCA	(AG)_13_	Ramsay *et al.* (2000)	172	Genomic DNA libraries	QTL abiotic stress	58	5.00	2.14	1.06	0.00	0.53	0.99	4.42
**HVM27**	HG	3H	56	GGTCGGTTCCCGGTAGTG	TCCTGATCCAGAGCCACC	(GA)_14_	Ramsay *et al.* (2000)	192	Genomic DNA libraries	QTL abiotic stress	64–55*	4.00	1.36	0.57	0.00	0.27	0.96	3.28
**HvDHN9**	HI	5H	N.D.	CATGGACAAGATCAAGGAGAAG	CCATTATTTATCTGTAGGAACGC	(AC)_6_	Becker and Heun (1995)	177	Barley genes	Dehydrin	58	4.00	3.24	1.23	0.01	0.69	0.98	3.77
**Bmac0040**	BO	6H	151	AGCCCGATCAGATTTACG	TTCTCCCTTTGGTCCTTG	(AC)_20_	Ramsay *et al.* (2000)	236	Genomic DNA libraries	QTL fusarium head blight resistance	58	13.00	8.68	2.30	0.01	0.89	0.98	10.23
**HVM14**	HD	6H	103	CGATCAAGGACATTTGGGTAAT	AACTCTTCGGGTTCAACCAATA	(CA)_11_	Ramsay *et al.* (2000)	158	Genomic DNA libraries	-	64–55*	4.00	1.91	0.81	0.00	0.48	1.00	2.99
**HVM62**	HB	3H	154	TCGCGACCAGACGAGAAG	AGCTAGCCGACGACGCAC	(GA)_11_	Ramsay *et al.* (2000)	251	Genomic DNA libraries	-	63–58*	5.00	1.88	0.94	0.00	0.47	0.99	4.13
**Bmac0154**	BD	1H	81	CTGGGTGATGAATAGAGTTTC	TATTCTTCAAAAGATGTTCTGC	(AT)_19_/(AC)_6_	Ramsay *et al.* (2000)	130	Genomic DNA libraries	QTL abiotic stress	58	6.00	2.36	1.13	0.01	0.58	0.98	4.72
**Ebmac0701**	EA	4H	76	ATGATGAGAACTCTTCACCC	TGGCACTAAAGCAAAAGAC	(AC)_23_	Ramsay *et al.* (2000)	149	Genomic DNA libraries	QTL abiotic stress	55	12.00	4.59	1.77	0.02	0.78	0.97	7.10
**HVM54**	HE	2H	103	AACCCAGTAACACCTGTCCTG	AGTTCCCTGACCCGATGTC	(GA)_14_	Ramsay *et al.* (2000)	159	Genomic DNA libraries	QTL days to heading, yield and fusarium head <@?show=[to]?>blight resistance	64–55*	6.00	2.22	0.97	0.01	0.55	0.98	3.63
**HvABA**	AB	*Unmapped*		CGCAGTACACCAAGGAGT	CGCATGCGTCTAGTGATT	(ACC)_5_	Ramsay *et al.* (2000)	221	Barley genes	Abscisic acid	58	2.00	1.04	0.09	0.00	0.03	1.00	1.45

*n_a_*, mean number of observed alleles; *n_e_*, mean number of expected alleles; *I*, Shannon’s information index; *H_o_*, observed heterozygosity; *H_e_*, expected heterozygosity; *F_is_*, inbreeding coefficient, *R_s_*, allelic richness.

All of the alleles were validated twice, using independent amplification of the same individuals. Indeed, a sub-set of genotypes that represented all of the alleles found in the whole sample were re-analysed following the described genotyping protocol.

### Statistical Analysis

The statistical analyses were designed to address the following aims:

1. Diversity analysis. Descriptive statistics on markers and populations were analysed and any differences were tested using non-parametric statistics.
2. Introgression. Population structure analyses were performed using AMOVA and STRUCTURE, to: (a) validate the approach using varieties of known hybrid origin (Micuccio, Pattyan and Zingaria); (b) analyse the overtime admixture between varieties and landrace populations, using non-parametric approaches to compare the data from the two collections of Sardinian landraces (1990 *vs*. 1999). Neutrality tests were used to identify loci putatively under the effects of selection, and to develop a putatively neutral dataset for the population structure and gene flow analysis. Indeed, gene flow is by definition a neutral phenomenon, and thus the careful validation of the assumption of neutrality is very important, to avoid biased estimation and to measure the relative role of gene flow and selection in determining the level of introgression.

#### Level of polymorphism

With the main aim of comparing the level of genetic diversity between the SL populations and the varieties (and within each sub-group), the following standard statistics were calculated to measure the genetic variation, using the POPGENE [24] and FSTAT [25] programmes: the number of alleles (*n_a_*), the effective number of alleles per locus (*n_e_* [26]), measures of allelic variation at a locus, the Shannon’s Information index (*I* [27]), the Levene [28] observed heterozygosity (*H_o_*), the Nei [29] unbiased expected genetic diversity (*H_e_*), the allelic richness (*R_s_* [30], a genetic variability estimate that takes into account the unbalanced sample sizes), and the intra-population fixation index (*F_is_* [31]), which is a measure of inbreeding. These were calculated for each SSR locus, for the whole sample, for each group (SL, VAR), and for each sub-group (SL90, SL99; Var2, Var6).

Moreover, considering the whole sample, the two main groups of SL/VAR, the 12 SL populations, the two variety populations (Var2/Var6), and the two years of collection (SL90/SL99), we calculated the number of total, private (allele present just in one population/group at various levels of subdivision: e.g., modern varieties, VAR *vs* landraces, SL) and shared alleles (excluding the rare alleles; frequency <0.05), by checking and counting manually in Microsoft Excel 2007.

#### Non-parametric test

To test the significances of the differences in *n_a_*, *n_e_*, *I*, *H_o_*, *H_e_*, *R_s_* and *F_is_*, we used the non-parametric Wilcoxon test, implemented in the JMP 7.0 software [32]. Significances were tested considering the two main groups (SL/VAR), the two years of landrace collection (SL90/SL99), the two types of varieties (Var2/Var6), all of these four sub-groups (SL90/SL99/Var2/Var6), the whole landrace population and the two types of varieties (SL/Var2/Var6), and the 12 SL populations. Non-parametric Wilcoxon tests were also used to test the differences in the average values of the structure membership coefficient, q, among the different samples.

#### Development of a “neutral dataset”

To obtain a putatively neutral dataset to be used for the population structure and gene flow analysis, we searched for loci showing signatures of selection in the SL dataset of 357 individuals and 11 polymorphic loci, using four different approaches [33]: (i) the Beaumont and Nichols [34] approach, further developed by Beaumont and Balding [35], and implemented in the FDIST2 software (http://www.rubic.rdg.ac.uk/~mab/software.html); (ii) the DetSel 1.0 approach [36], [37]; and (iii) *lnRH* and *lnRV* tests, implemented in Microsatellite Analyser (MSA) software, version 4.05 [38]; (iv) the ‘detection of loci under selection from the F-statistic’ procedure, implemented in Arlequin, version 3.5 [39], [40].

All of the methods were applied as described in [5], with the exception of the ‘detection of loci under selection from the F-statistic’ procedure. This is an approach similar to that of FDIST2, but this method allows the use of a different mutation model, the step-wise mutation model, specific for microsatellite data, and gives the possibility to define a hierarchical island model that might help to reduce the number of false positives [41]. In this case, 200,000 simulations were run, testing different combinations of demes per groups, and groups.

The loci identified as putatively under selection using these methods were discarded, and a neutral dataset was obtained and used for further analysis.

#### Spatial autocorrelation analysis

Spatial Genetic Software, version 1.0 d [42], was used in the SL dataset to test the spatial autocorrelation between genetic distances and spatial-variable distance matrices (geographical, latitudinal or altitudinal), based on single individuals. These calculations were carried out using Moran’s I [43], [44] for spatial distance classes, the dimension of which was 20 km for geographical distances (7 classes), 15 km for latitudinal distances (7 classes), and 34 m for altitude differences (10 classes). The sizes and numbers of the classes were fixed in order to guarantee at least 1,000 pairs of data points in each class for statistical significance and to account for biological meaning. The significances of the observed average Moran’s I values were assessed by comparing them with the corresponding values derived by randomly permuting (500 replicates) the multilocus genotypes over the spatial coordinates, and in our case, the 99% confidence intervals were estimated. Spatial correlation analysis was also computed using rainfall data (mm) for each location, as 10 classes of 34 mm of annual rainfall. We obtained the Moran’s I correlograms over all of the 11 polymorphic SSR loci.

The GenAlEx software, version 6 [45], was used to test the association between the matrices of genetic and geographical distances using the Mantel test based on single individual comparisons [46]. With the aim of finding possible correlations with other important spatial and environmental variables, the Mantel test was also carried out with latitudinal, altitudinal and rainfall data matrices.

#### Population structure

The whole dataset was used for an investigation of the population structure. This comprised 416 individuals, as all of the SL populations and all of the VAR populations, analysed with 12 SSR markers. The population structure was examined using the assignment method implemented in the STRUCTURE software, version 2.2 [47], which infers the number of clusters, K (populations), that might be present in a sample, by comparing the posterior probability for different numbers of putative populations specified by the user, and it assigns individuals to these clusters, giving their percentages of membership (q). Twenty independent runs for each K (from 1 to 15) were performed using 30,000 Markov Chain Monte Carlo (MCMC) repetitions and 30,000 burn-in periods, using no prior information, and assuming correlated allele frequencies and admixture. The number of clusters (K) was estimated by computing the *ad-hoc* statistic ΔK, based on the rate of change in the log probability of the data between successive K values [48]. Based on the statistic ΔK, the most likely number of clusters (K) was two, as this number maximised the ΔK parameter [48]. A final run at 100,000 MCMC repetitions and 100,000 burn-in periods was performed. The output of the software gives the percentages of membership for the K clusters for each individual. We then computed the average percentages of membership (q) for each of the inferred K clusters of the two main groups of individuals (SL, VAR).

To infer the population structure in a neutral context, we also performed population structure analysis on the whole dataset of the 416 individuals using the 10 SSR markers identified as ‘neutral’ (nine plus one that was monomorphic, HvABA, among the SLs), out of the 12 polymorphic SSRs in the whole dataset (see section ‘Development of a “neutral dataset”). In this case, based on the statistic ΔK, the most likely number of clusters (K) was three, and the software was run using the final run parameter set described above.

#### Population differentiation

Partitioning of the total genetic variance was obtained by applying the AMOVA framework [49], implemented in the Arlequin software, version 3.5 [39]. The divergence between the SL and VAR populations, and between the SL years of collection (SL90/SL99), were estimated for each of the SSR loci, and as average overall loci, using *F_ST_* [50]. Similarly, the pairwise genetic divergence among SL90, SL99, Var2 and Var6 and among the 12 SL populations were computed. All of the pairwise comparisons were significant (*P*<0.001).

## Results

When considering the whole sample of SL and VAR, all of the 12 SSR markers were polymorphic: a total of 67 alleles were detected, and the number of alleles per locus ranged from two (HVMSIP1A and HvABA) to 13 (Bmac0040), with an average of 5.6 alleles per locus (Table 2). Considering only the SL populations, the HvABA locus was monomorphic; for the remaining 11 polymorphic loci, a total of 56 alleles were identified, and the number of alleles per locus varied from two (HVMSIP1A) to 13 (Bmac0040), with an average of 4.7 per locus. The missing data (null phenotypes) represented 1.8% of the whole dataset, with five markers with a frequency of the null phenotype <1.5%; the remaining seven markers ranged from 1.7% (Bmac0273 and HVM54) to 3.8% (BMS40). Null phenotypes can originate through failed PCR amplifications or can be homozygous for the null alleles.

The statistics of the genetics are listed in Table 3. SL90 and SL99 showed the same number of polymorphic loci (11 loci out of the 12 SSRs used, 91.7%) and the polymorphism within the populations varied from 75.0% (9 loci) for SAF, STU and VI, to 91.7% (11 loci) for COR, CUM, PIR, SEN, SIS3 and SOR. The SL overall genetic diversity (*H_e_*) was 0.46, which did not vary significantly between SL90 (0.46) and SL99 (0.45), and it ranged from 0.32 for STU, to 0.44 for CUM. The SL overall allelic richness (*R_s_*) was 4.09, which did not vary significantly between SL90 (3.48) and SL99 (3.56), and which ranged from 3.29 for CUM and SEN, to 2.57 for VI. The SL observed heterozygosity (*H_o_*) was 0.01, which did not vary between SL90 and SL99 (0.01 for both years), and which ranged from 0.00 for ORO and VI, to 0.02 for CUM. For the comparisons tested (within SL and SL90/SL99), there were no significant differences for any of the statistics estimated.

**Table 3:** Summary statistics calculated for each population (12 SL, Var2 and Var6), for each group and sub-group (SL and VAR, SL90 and SL99,) and for the whole sample.

Population	Polymorphic loci (n)	Polymorphic loci (%)	S	n_a_	n_e_	I	H_o_	H_e_	R_s_	F_is_	Total allele (n)
**VI**	9	75.0	58	2.67	1.18	0.59	0.00	0.34	2.57	0.99	32
**SAF**	9	75.0	59	3.08	2.17	0.70	0.01	0.40	2.96	0.97	37
**SEN**	11	91.7	60	3.42	2.18	0.79	0.01	0.43	3.29	0.99	41
**SIS3**	11	91.7	56	2.83	1.98	0.68	0.01	0.39	2.78	0.98	34
**ORO**	10	83.3	60	3.33	2.11	0.77	0.00	0.43	3.18	1.00	40
**N2**	10	83.3	56	3.08	2.15	0.74	0.01	0.43	3.01	0.99	37
**SL90**	11	91.7	349	4.58	2.49	0.87	0.01	0.46	3.48	0.99	55
**COR**	11	91.7	59	3.33	1.97	0.75	0.01	0.42	3.20	0.97	40
**NXM**	10	83.3	59	3.33	2.01	0.73	0.01	0.40	3.20	0.98	40
**STU**	9	75.0	60	2.83	1.64	0.56	0.01	0.32	2.70	0.97	34
**SOR**	11	91.7	59	3.25	2.27	0.74	0.01	0.40	3.15	0.99	39
**PIR**	11	91.7	60	3.08	1.78	0.64	0.01	0.36	2.94	0.99	37
**CUM**	11	91.7	58	3.50	2.22	0.79	0.02	0.44	3.29	0.96	42
**SL99**	11	91.7	355	4.17	2.44	0.86	0.01	0.45	3.56	0.97	50
**SL**	**11**	**91.7**	**704**	**4.67**	**2.51**	**0.88**	**0.01**	**0.46**	**4.09**	**0.98**	**56**
**Var2**	11	91.7	39	3.42	2.23	0.89	0.00	0.51	3.41	1.00	41
**Var6**	11	91.7	74	3.75	2.03	0.84	0.00	0.47	3.49	1.00	45
**VAR**	**12**	**100.0**	**113**	**4.08**	**2.23**	**0.94**	**0.00**	**0.51**	**4.08**	**1.00**	**49**
**All**	**12**	**100.0**	**817**	**5.58**	**2.75**	**1.02**	**0.01**	**0.51**	**4.97**	**0.98**	**67**

The SL population codes are in **Table 1**.

*S*, sample size; *n_a_*, mean number of observed alleles per locus; *n_e_*, mean number of expected alleles per locus; *I*, Shannon’s information index; *H_o_*, observed heterozygosity; *H_e_*, expected heterozygosity; *R_s_*, allelic richness; *F_is_*, intra-population fixation index.

In the VAR sample, all of the markers were polymorphic, and Var2 and Var6 showed the same number of polymorphic loci (11 loci, 91.7%). The VAR overall genetic diversity (*H_e_*) was 0.51, which did not vary significantly between Var2 (0.51) and Var6 (0.47); the allelic richness (*R_s_*) was 4.08, which did not vary significantly between Var2 (3.41) and Var6 (3.49), and the VAR observed heterozygosity (*H_o_*) was 0.00.

Overall, based on the standard diversity estimations used, we observed that the six-rowed barley landrace populations from a relative restricted area such as the island of Sardinia have the same level of diversity detected in the large collection of modern varieties including both two and six row types. Moreover, our data show that some farmers’ fields (e.g., SEN and CUM) maintain a level of diversity comparable to that of the two-rowed or six-rowed varieties.

No significant differences in the statistics estimated for any of the comparisons were detected, with the exception of the intra-population fixation index (*F_is_*) and the observed heterozygosity (*H_o_*), which were significantly different (*P*<0.001) for the comparisons of SL/VAR, SL90/SL99/Var2/Var6 and SL/Var2/Var6, with the landraces (SL, SL90 or SL99) having values of *H_o_* that were always higher, and of *F_is_* that were always lower, than the varieties (VAR, Var2 or Var6) (e.g., SL *H_o_* = 0.01 and VAR *H_o_* = 0.00; SL *F_is_* = 0.98 and VAR *F_is_* = 1.00; Table 3), suggesting the potential for hybridisation to occur in the landrace group.

### Development of a “Neutral Dataset”

We test our dataset for neutrality to exclude loci putatively under selection (or because of genetic sweep) when inferring the population structure of the SLs and estimating the gene flow between VAR and SL; we decided to keep only those markers for which the null hypothesis of neutrality was never rejected by any of the methods used.

Considering the results of the tests of signatures of selection (i.e., Figure S1), and adopting a conservative perspective for Type II error, two loci, (HvDHN9 and HVM54) were thus discarded for the subsequent population structure and gene-flow analysis.

### Spatial Structure and Autocorrelation Analysis

For the 11 polymorphic loci, the Mantel test was applied to the matrix of Nei’s unbiased genetic distances with the matrix of geographical, latitudinal and altitudinal distances, and the rainfall data matrix (Table S2), to highlight possible structures in the genetic variation. The genetic distance-latitude showed the highest correlation between the two matrices (r = 0.29; *P*<0.01), while lower correlations seen for the genetic–geographical distance (r = 0.25; *P*<0.01).

**Figure 1:**
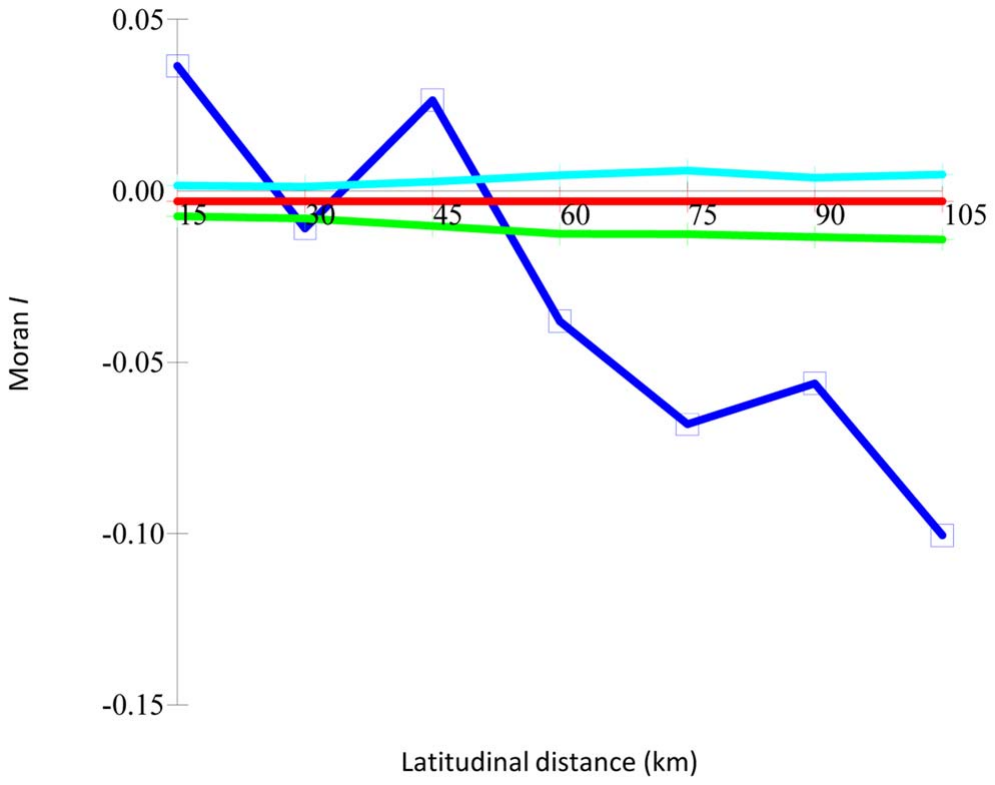
Autocorrelation analysis for latitudinal distances *versus* genetic distances using the 357 SL genotypes and 11 polymorphic SSR loci. Light blue line, upper limit, and green line, lower limit, of 99% probability envelopes; dark blue line, observed data.

Using all of the 11 loci, spatial autocorrelation analysis showed that there was a relationship between the genetic and latitudinal distances. Indeed, individuals collected at the same latitude (including those from the same collection site) showed higher similarities than those at different latitudes (Figure 1): starting from positive Moran’s I values, the degrees of correlation decreased, and negative I values were found for the 45 km class (3^rd^) and above. A less marked trend was found for the genetic-geographic distances and no significant trends found for the altitude and rainfall levels (data not shown).

### Landraces and Varieties

#### Population structure

By STRUCTURE analysis on the whole dataset of 416 genotypes (SL+VAR), and using all of the 12 SSR loci, we identified K = 2 clusters (data not shown), with all of the SL populations belonging to cluster 1 (average SL q_1_ = 0.99), and all of the varieties belonging to cluster 2 (VAR q_2_ = 0.95; excluding the three control varieties Micuccio, Pattyan and Zingaria, VAR* q_2_ = 0.99).

**Figure 2:**
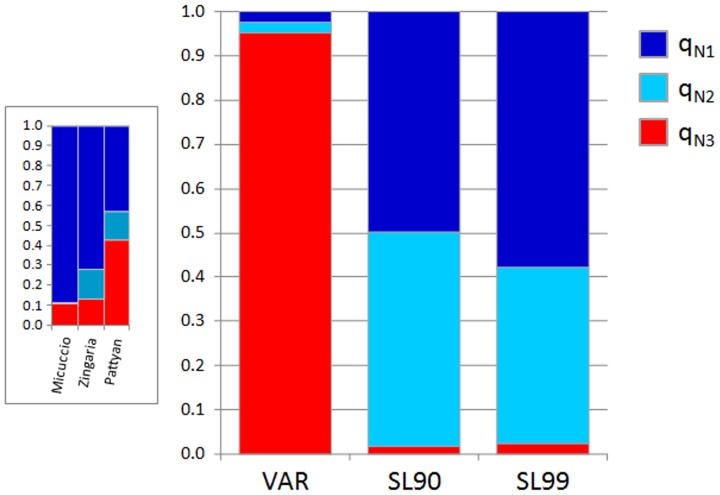
Membership percentages for the SL and VAR population groups, using neutral dataset markers. For VAR, the three varieties considered as controls (Micuccio, Zingaria and Pattyan, showing genotypes from Italian landrace population in their pedigrees) were excluded and their membership percentages are shown separately in the box on the left. **q_N1_**, neutral dataset membership to cluster 1; **q_N2_**, neutral dataset membership to cluster 2; **q_N3_**, neutral dataset membership to cluster 3.

**Figure 3:**
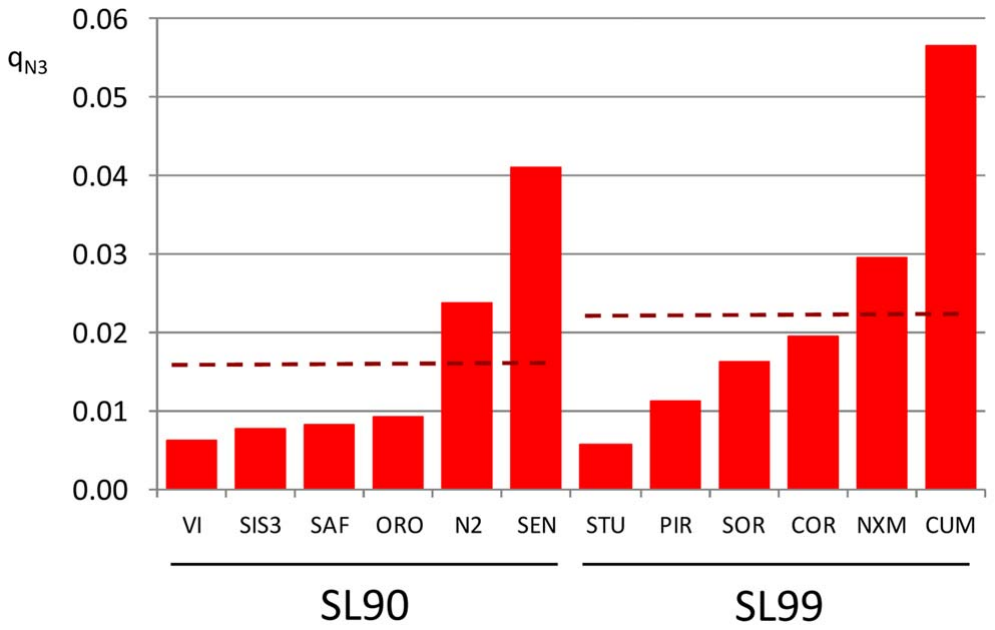
Neutral dataset membership to cluster 3 (q_N3_) for the Sardinian 1990 (SL90) and 1999 (SL99) landrace populations using neutral dataset markers. SL populations are ordered according the increasing q_N3_; dashed dark red lines, mean q_N3_ for each year of collection.

We performed the STRUCTURE analysis using the set of 10 loci (nine plus one that was monomorphic among the SL, but polymorphic in VAR), excluding the two loci putatively under selection. In this case, using the Evanno method, we identified K = 3 groups (Figure S2). The average percentages of membership were computed: q_N1_ for cluster 1, q_N2_ for cluster 2, and q_N3_ for cluster 3. The results are shown in Figure 2. All of the varieties were assigned to cluster 3 (VAR q_N3_ = 0.91; VAR* q_N3_ = 0.95). The SL populations were assigned to clusters 1 and 2, with intermediate values of membership of SL q_N1_ = 0.44 and SL q_N2_ = 0.54, and with a very low value of membership for cluster 3 (SL q_N3_ = 0.02). The SL99 group showed a slightly higher, but significant (*P*<0.05), percentage of individual membership to the varieties cluster (SL99 q_N3_ = 0.023), compared to SL90 (SL90 q_N3_ = 0.016) (Figure 3), with an increment of about 50%. Considering the SL populations, we observed significant (*P*<0.01) differentiation for the values of membership to the varieties cluster (q_N3_). Taking into account the single individuals, we noted that two genotypes, one from the CUM population (SL99 Cuggia Manna population, northern Sardinia) and one from the NXM population (SL99 Nuraxi Mannu population, southern Sardinia) were admixed and had a value of membership to the varieties cluster of 0.60 and 0.40 respectively. Moreover, 23 individuals from the SL90 and SL99 populations showed q_N3_≥0.05.

#### Gene flow and introgression

No private alleles were found in any of the SL populations; the two-rowed varieties showed two private alleles. Comparing all of the SL and the VAR groups, we found 9 SL private alleles, and 11 VAR private alleles. Out of the total of 67 alleles detected in the whole sample, the SL populations, SL90 and SL99, shared the highest proportion of alleles (49 alleles, 73%) (Table S3). The SL population and VAR shared 38 alleles (57%; 35 with VAR*); similar results were obtained when these two years of collection were considered separately: SL90 and SL99 shared 37 alleles (55%) and 35 alleles (52%), respectively, with the VAR group; moreover, the SL population shared 30 and 36 alleles with Var2 and Var6 (30 with Var6*), respectively. Var2 and Var6 shared 37 alleles.

Table 4 gives the results of the AMOVA analysis. Considering only the SL populations, there was no differences in the allele frequencies between the years of collection (SL90 and SL99); most of the variation was found within populations (i.e., within a farmer’s field; 84.1%, *P*<0.0001), and just 14.1% (*P*<0.0001) was observed between populations. As expected, the within individuals component (due to the level of heterozygosity) was very low (1.8%, *P*<0.0001).

**Table 4:** Analysis of the molecular variance using SSR markers, performed at different levels.

Source of variation	df	Sum of squares	Variance component	% variation	*P*
Among years (SL90/SL99)	1	17.8	0.0	**0.0**	ns
Among populations within years	10	277.2	0.4	**14.1**	**
Among individuals within populations	343	1614.1	2.3	**84.1**	**
Within individuals	355	17.0	0.1	**1.8**	**
**Total**	**709**	**1926.1**	**2.8**		

ns, not significant;

*P*<0.0001.

SL90, Sardinian landraces collected in 1990; SL99, Sardinian landraces collected in 1999.

The lowest *F_ST_* was between SL90 and SL99 (*F_ST_* = 0.02); the highest *F_ST_* were between SL90 and Var2 (*F_ST_* = 0.33) and SL99 and Var2 (*F_ST_* = 0.34). The overall divergence between the SL and Var2 groups was *F_ST_* = 0.33. The comparisons between SL90 and SL99 with Var6 showed intermediate values of *F_ST_* (*F_ST_* = 0.26 in both cases; overall SL *vs* Var6, *F_ST_* = 0.25), even with the three control varieties excluded from the Var6 group (Micuccio, Pattyan and Zingaria) (SL90 *vs*Var6* and SL99 *vs* Var6*, *F_ST_* = 0.28; overall SL *vs*Var6*, *F_ST_* = 0.27) (Table S4). The comparisons between Var2 and Var6 (or Var6*) gave an *F_ST_* of 0.14.

## Discussion

To the best of our knowledge, the present study is the first that directly compares the population diversity of barley landraces, even at a field (within population) level, with a representative pool of barley varieties. In particular, using SSRs, 12 barley landrace populations from Sardinia from two collections that spanned 10 years, and 59 commercial modern varieties were analysed.

### Introgression

The aim of this study was to compare the SL population (collected in two years that were a decade apart) with a large and representative set of two-row and six-row modern barley varieties, to estimate the extent of gene flow and to determine the level of introgression. The VAR were chosen to include almost all of the commercial varieties grown in Italy over three decades (from 1970 to 2000). Introgression was analysed based on the population structure revealed by SSR loci that were tested for neutrality, to take into account the effects of selection. Indeed, the effect of migration between populations is to homogenise the allelic frequencies due to the introgression of alleles from the donor to the recipient population, and the level of introgression can be highly affected by selection or because of hitchhiking [51], [52], which in an autogamous species such as barley might be substantial, because of the slow decay of the linkage disequilibrium [53], [54]. Thus, if the analysis of the population structure is to be used to infer the level of migration, it is crucial to carefully consider that the inference is based upon the assumption of neutrality. Thus we searched for the signature of selection among the molecular markers used in this study and developed a putative neutral SSR dataset.

Our analysis clearly shows that the SL populations constitute a distinct gene pool from those of both the two-row and six-row barley varieties. The results obtained for the three varieties derived from a cross between genotypes extracted from Italian landraces and modern varieties and included in this study as references, Micuccio, Zingaria and Pattyan, indicate that the SLs share (at least partially) a common genetic background with other Italian barley landraces. Indeed, these three varieties were all found to be admixed (Pattyan) or assigned to the SL clusters with high levels of membership (from 0.90 to 0.87) (Figure 2). This result also clearly indicates that the approach adopted here to study gene flow is a powerful one for the detection of genotypes derived from hybridisation and introgression between varieties and landraces. Thus we were able to estimate the level of introgression from the admixture coefficient obtained from the STRUCTURE analysis.

The most important result obtained in this study is the occurrence of an average low (but significant) and population-dependant level of introgression from VAR to SL. This consideration is based on the following observations:

1. Two SL genotypes (out of 357) were admixed (0.4< q_N3_<0.60);
2. Significant differences were observed among the SL populations for the average assignment coefficient q_N3_ (the VAR cluster);
3. The collection of 1999 showed a significantly larger admixture coefficient (q_N3_) compared to the 1990 collection.

Indeed, a higher level of introgression observed after 10 years is expected if introgression is a relevant phenomenon. In modern agriculture, if gene flow is present, it is unidirectional (unless breeders use landraces as a source of germplasm), i.e., from the modern varieties (where the seed is multiplied in isolation by the seed company) to the landraces (where the seed is multiplied by farmers); thus, over time, the level of introgression is expected to increase. In particular, in the case of Sardinia, the spread of commercial varieties started approximately in the 1980’s, and thus the observation of an increased level of introgression in our case spans the 10-year period that occurred from 10 to 20 years after the spread of the commercial varieties.

However, the overall level of introgression is very low, which is in agreement with the breeding system of barley (strictly autogamous) and by the occurrence of strong selection against short plants in bulk populations [55]. This latter reduces the fitness of the first generation hybrids, and as a consequence, limits the establishment of alleles from modern varieties. Indeed, in cereals, modern varieties have a reduced plant height compared to landraces because of the presence of dwarf and semi-dwarf genes that render the modern plants resistant to lodging [56], [57]. Moreover, considering the barley breeding system and the short pollen dispersal (up to 60 m [58], [59]), gene flow is most likely to be mainly due to seed flow followed by hybridization, rather than pollen flow between different fields. For instance, many farmers harvest their fields using contractors, and thus seed flow can occur by the use of the same combine harvester among farmers growing landraces and varieties.

Moreover, although the observed heterozygosity within SL is very low, it is significantly higher than within the VAR groups, which suggests that within the fields of the landrace, there was indeed the potential for hybridisation to occur (as also, consequently, for the potential for introgression).

The low level of introgression identified is also population dependent: the gene flow from varieties to landraces was limited, and the uneven level of introgression detected is most likely to be due to the individual farmers’ agronomic practices (e.g., seed source, land preparation, sowing, harvesting), and is not a widespread and uniform phenomenon. A similar result was found by Bitocchi *et al*. [5] in maize landraces.

### Level and Structure of Diversity in Barley Landraces

Our data show that these 12 barley landrace populations, that all belong to the same six-rowed landrace “S’Orgiu Sardu”, that has been conserved *in situ* and that originated from a relatively restricted geographical area, have the same level of diversity that occurs in the pool of commercial varieties from both two-row and six-row types that originated from different breeding programmes all over Europe. This is in agreement with what has been observed in different species where such comparisons have been made directly [5] or indirectly [8], [23]. This is further confirmation that *in-situ* conservation of landrace populations is an effective and valuable strategy to preserve crop genetic resources [7].

Moreover, as found in various other studies, we have also confirmed here that landraces are variable populations that are constituted by a large number of different genotypes (for review, see [60]), also when strict selfing species such as barley are considered [61]. Indeed, the within population component of genetic variance in Sardinian barley landraces was 0.14 using SSRs (present study), and varied from 0.11 (RAPD [15]) to 0.18 (S-SAP [17]) using molecular markers, while both for isozymes and morphological markers, *F_ST_* was 0.16 [15]. Similar observations have been made for other barley landraces, even if a larger, between-population component was seen when wider ecological and geographical sampling was considered (in Syria and Jordan [62]; in North Shewa, Ethiopia [23]). Our study is the first that compares the within population (field) diversity of barley landraces with a representative sample of barley varieties. For this reason we were able to show that the within population diversity of barley landraces from Sardinia is comparable to that observed within one of the two major gene pools of barley modern varieties (two row *vs.* six row). As shown in other studies on SL, the level of diversity within a population is also associated with relevant agronomic traits [63] and to variations in salt tolerance [64], [65]. These results are in agreement with other studies on barley landraces, where within-population variation has been seen for many important traits, including disease resistance [66], [67]. The occurrence of such diversity within a population is thus very important for crop improvement. Moreover, these studies also suggest a putative adaptive role for such a component [1], [66], even if further studies need to be conducted to obtain a more robust indication.

Landrace populations are considered to be locally adapted to their environments [68]. However, very little direct evidence supports this statement [1]. Tanto Hadado *et al*. [23] showed that molecular variation between populations is associated with selection for adaptation to variable altitudes. From the significant correlation between the genetic and geographic distances of the landrace populations of Thai rice, an isolation by distance (IBD) [69] population structure was suggested, which indicates that these landraces represent a dynamic genetic system that responds to natural and artificial selection, which promotes the local adaptation of landrace populations [70]. In our case, the significant correlations among genetic and geographic and latitudinal distances (Mantel tests) and the results of the Spatial Autocorrelation analysis also indicate an IBD model of population differentiation, where distances appear to contribute to isolation and genetic divergence, with populations at greater distance more genetically different than populations close to each other; this effect is stronger for latitudinal (North/South cline), than for geographical distances. Our data, as with those for Thai rice, are in agreement with the hypothesis that landraces have the potential for adaptation to their environment.

### In-situ Conservation

Along with another study on different species [5], our study demonstrates that the conservation of landrace populations *in situ* is compatible with the coexistence with industrial agriculture and also with genetically modified varieties. Indeed, landraces maintain their identity and diversity even in the presence of gene flow that also occurs in strict selfing species; here, we showed that there is a low, but significant (comparing VAR with SL), level of heterozygosity, which indicates that hybridisation between individuals is an active phenomenon in barley landraces that contributes to the process of adaptation of SL. The data presented here also indicate that gene flow can be drastically limited by the adoption of appropriate practices; this consideration arises from the observed variation in the level of introgression between populations that overcome their geographical distribution, and from the similar results obtained on maize landraces from central Italy [5].

Thus, the building of appropriate dynamic *in-situ* conservation is a very important strategy to develop and maintain crop germplasm, and to preserve its potential for adaptation, particularly when the within population variation is the focus of the conservation programme. With dynamic *in-situ* management, populations are indeed maintained, which allows the action of evolutionary forces on the cultivated species and the adaptation to agro-ecological changes [55], [71]; the aim is to conserve wide within species adaptive variability, rather than specific alleles at a locus, or some cultivars that are already genetically fixed.

Moreover, the conservation of such materials can also be very important to identify genes and loci with adaptive values, through the exploitation of three different strategies: (i) association genetics [72]; (ii) population genetics [73]; and (iii) ecological variation analysis [74], [75]. Landraces indeed offer the opportunity to exploit their unique proprieties given by the levels of genetic variation and linkage disequilibrium, the population structure, and the strong and historical link with their environments and agro-ecological conditions [53], [60], [61].

Overall, this indicates the need to develop a systematic programme of dynamic *in-situ* conservation for different crop species [55].

## Supporting Information

Figure S1 ***F_ST_ versus*** **heterozygosity estimates, obtained using Arlequin, version 3.5, assuming a hierarchical model of migration.** Dashed blue line, 95% confidence interval; dashed red line, 99% confidence interval; solid grey line, median; filled blue circles, loci significant at the 5% level; red filled circles, loci significant at the 1% level; empty circles, putatively neutral loci.
(PDF)

Figure S2 **Average** ***lnlikelihood*** **values over 20 runs for increasing K values, from 1 to 15, using the neutral markers dataset in the whole sample of 416 individuals, and ΔK values over 20 runs for increasing K values, from 2 to 15.** Green arrow, number of cluster (K) that maximises the ΔK parameter.
(PDF)

Table S1 **Details of the 59 barley varieties (Var) used in this study, giving: name, spike type, growth class, country and year of origin, and pedigree information.** ^(1)^ Spike row type: two-rowed, 2R; six-rowed, 6R. ^(2)^ Growth class: Spring type, S; Winter type, W; Intermediate type, I. Country abbreviation code: Belgium, BE; France, FR; Germany, DE; Italy, IT; Netherlands, NL; Sweden, SE; United Kingdom; GB.
(XLSX)

Table S2 **Results of the Mantel test performed according to geographical distance (GeoDist), latutudinal distance (Latit), altitude (Altit) and rainfall (Rainf).** ns, not significant; * P<0.05, ** P<0.01.
(XLSX)

Table S3 Shared alleles between SL (SL90, SL99), Var2, Var6 (Var6*) and VAR (VAR*).
(XLSX)

Table S4 **Pairwise** ***F_ST_*** **between SL (SL90, SL99), Var2 and Var6.**
(XLSX)
