# Supplementary material for: Population Structure of Barley Landrace Populations and Gene-Flow with Modern Varieties
Source: PLoS One. 2013 Dec 27;8(12):e83891. doi: 10.1371/journal.pone.0083891 (PMC3873955; doi:10.1371/journal.pone.0083891)
Supplement: Figure S1 — FST versus heterozygosity estimates, obtained using Arlequin, version 3.5, assuming a hierarchical model of migration. Dashed blue line, 95% confidence interval; dashed red line, 99% confidence interval; solid grey line, median; filled blue circles, loci significant at the 5% level; red filled circles, loci significant at the 1% level; empty circles, putatively neutral loci. (PDF) [file pone.0083891.s001.pdf]

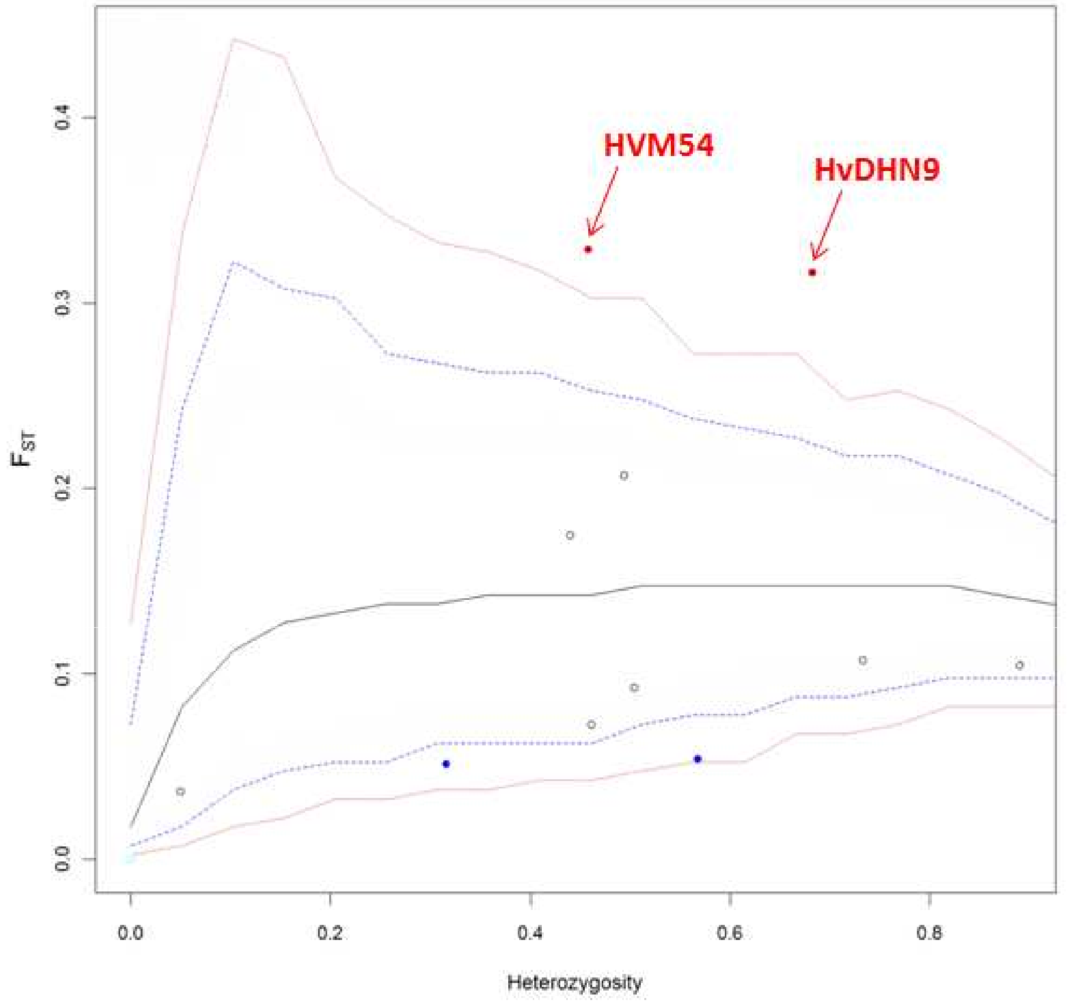

Figure S1. F_{ST} *versus* heterozygosity estimates, obtained using Arlequin, version 3.5, assuming a hierarchical model of migration.

Dashed blue line, 95% confidence interval; dashed red line, 99% confidence interval; solid grey line, median; filled blue circles, loci significant at the 5% level; red filled circles, loci significant at the 1% level; empty circles, putatively neutral loci.
