# Supplementary material for: Population Structure of Barley Landrace Populations and Gene-Flow with Modern Varieties
Source: PLoS One. 2013 Dec 27;8(12):e83891. doi: 10.1371/journal.pone.0083891 (PMC3873955; doi:10.1371/journal.pone.0083891)
Supplement: Figure S2 — Average lnlikelihood values over 20 runs for increasing K values, from 1 to 15, using the neutral markers dataset in the whole sample of 416 individuals, and ΔK values over 20 runs for increasing K values, from 2 to 15. Green arrow, number of cluster (K) that maximises the ΔK parameter. (PDF) [file pone.0083891.s002.pdf]

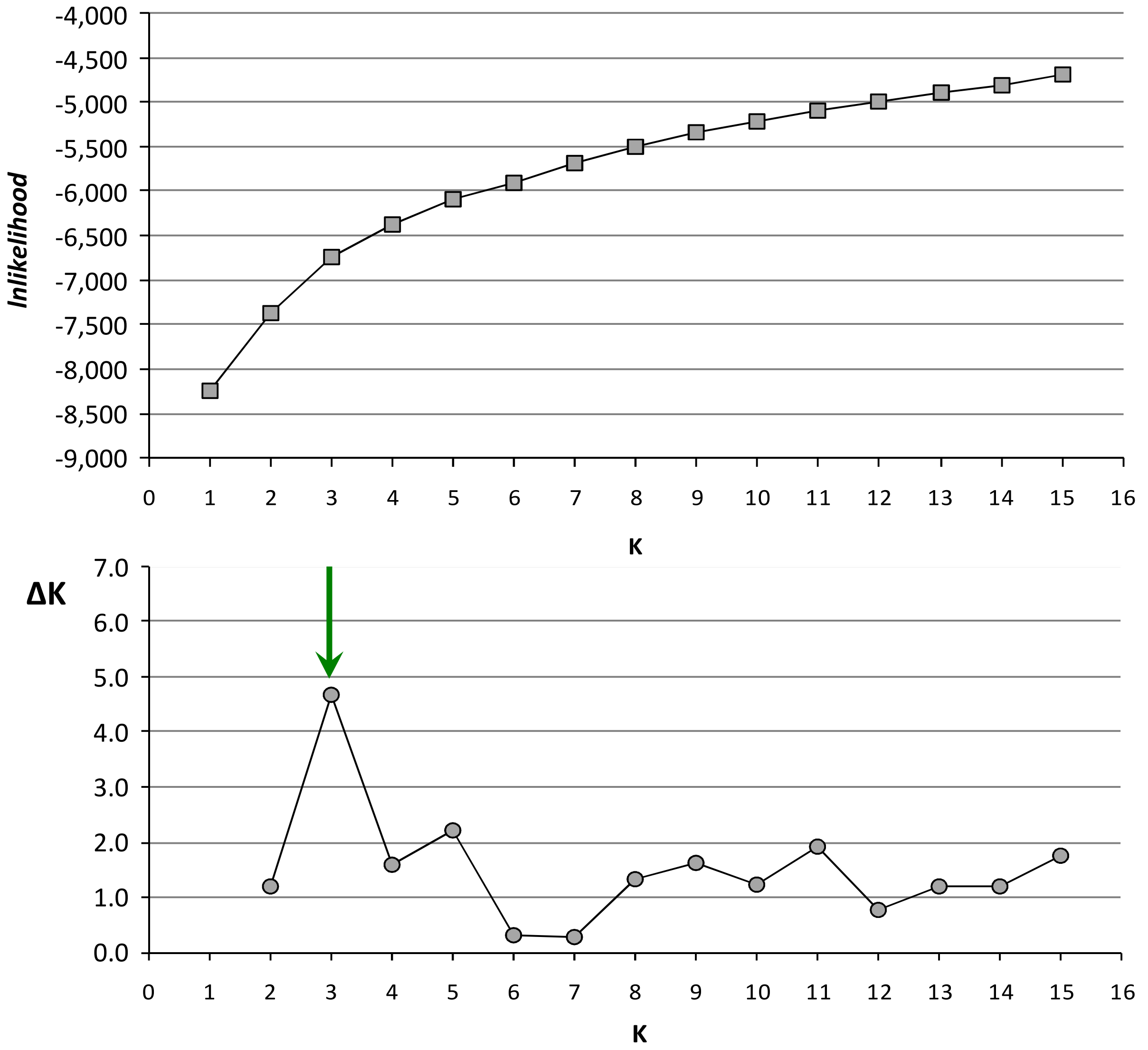

Figure S2. Average *lnlikelihood* values over 20 runs for increasing K-values, from 1 to 15, using the neutral markers dataset in the whole sample of 416 individuals and ΔK values over 20 runs for increasing K-values, from 2 to 15. Green arrow identify the number of cluster (K) that maximized the ΔK parameter.
